# Transcriptional signatures associated with waterlogging stress responses and aerenchyma formation in barley root tissue

**DOI:** 10.1093/aob/mcaf104

**Published:** 2025-06-18

**Authors:** Orla L Sherwood, Rory Burke, Jennifer O’Rourke, Conor V Whelan, Frances Downey, Louise Ryan, Eoin F McCabe, Zixia Huang, Carl K Y Ng, Paul F McCabe, Joanna Kacprzyk

**Affiliations:** School of Biology and Environmental Science, University College Dublin, Belfield, Dublin 4, Ireland; School of Biology and Environmental Science, University College Dublin, Belfield, Dublin 4, Ireland; School of Biology and Environmental Science, University College Dublin, Belfield, Dublin 4, Ireland; School of Biology and Environmental Science, University College Dublin, Belfield, Dublin 4, Ireland; School of Biology and Environmental Science, University College Dublin, Belfield, Dublin 4, Ireland; School of Biology and Environmental Science, University College Dublin, Belfield, Dublin 4, Ireland; School of Biology and Environmental Science, University College Dublin, Belfield, Dublin 4, Ireland; School of Biology and Environmental Science, University College Dublin, Belfield, Dublin 4, Ireland; School of Biology and Environmental Science, University College Dublin, Belfield, Dublin 4, Ireland; School of Biology and Environmental Science, University College Dublin, Belfield, Dublin 4, Ireland; School of Biology and Environmental Science, University College Dublin, Belfield, Dublin 4, Ireland

**Keywords:** Barley, root transcriptomics, aerenchyma, waterlogging, flooding

## Abstract

**Background and Aims:**

The frequency of extreme precipitation events is predicted to increase owing to climate change, leading to soil waterlogging and crop yield losses, particularly in the case of susceptible species, such as barley (*Hordeum vulgare*). Aerenchyma formation is a key morphological adaptation to waterlogging stress and hypoxic conditions; however, its genetic regulation in barley remains largely unresolved. The aim of this study was to address this knowledge gap and characterize the transcriptional signatures associated with the waterlogging stress response and aerenchyma formation in barley roots.

**Methods:**

Two barley cultivars (Franklin and Yerong) were subjected to waterlogging stress, followed by analysis of phenotypic traits, including root aerenchyma formation, and transcriptomic profiling of root tissue. Differential gene expression analysis and gene regulatory network construction were carried out using generated RNA-sequencing datasets.

**Key Results:**

Performed analyses identified genes transcriptionally responsive to 24 and 72 h of waterlogging in both cultivars and highlighted metabolic adaptations, regulation of reactive oxygen species signalling and management of stress responses as key elements of the waterlogging response in barley roots. Large intra-individual variation was observed for root aerenchyma formation. This variation was exploited to identify 81 candidate aerenchyma-associated genes and ascertain pathways involved in aerenchyma formation. Furthermore, network analyses suggested that the DNA damage response gene *DRT100* and the cell wall-modifying genes *XTH16* and *XTH15* are regulatory hub genes in aerenchyma formation.

**Conclusions:**

This study provides new insights into transcriptional signatures associated with waterlogging responses and aerenchyma formation in barley roots. The identified candidate aerenchyma-associated genes offer new targets for future research and breeding efforts aimed at enhancing waterlogging tolerance in this crop species.

## INTRODUCTION

Waterlogging is a severe abiotic stress. Owing to lower oxygen diffusion rates in water than in air, the oxygen availability to submerged plant tissues is reduced, leading to an average of 33 % yield loss in affected crops (Tian *et al*., 2021). Altered rainfall patterns are consequences of climate change, resulting in an increased risk of flooding in many areas (Bailey-Serres *et al*., 2012; Westra *et al*., 2014). It has been projected that extreme precipitation events will occur 32 % more frequently by 2100 (Thackeray *et al*., 2022); therefore, development of flood-tolerant crop cultivars is urgently required to protect food security. Plant tolerance to flooding stress differs widely across species: wetland plants, such as rice, generally exhibit high tolerance, whereas many other key crops, including barley (*Hordeum vulgare*), show sensitivity to even short-term waterlogging, further underscoring the need to develop tolerant varieties (Smirnoff and Crawford, 1983; De Castro *et al.*, 2022). In barley (*H. vulgare*), the focus species of this study, yield losses attributable to waterlogging can reach ≤70 %, depending on the stress duration, soil type, temperature and the developmental stage when stress is imposed (Pang *et al*., 2004; de San Celedonio *et al*., 2016; Liu *et al*., 2020). Given that barley is the fourth most important cereal crop globally (Tricase *et al*., 2018), it is crucial to elucidate the molecular mechanisms underlying waterlogging stress adaptation in this species to inform development of tolerant germplasm. Indeed, there is extensive evidence for genetic regulation of waterlogging tolerance in plants, including barley, suggesting a high potential to breed for improved waterlogging resilience. For example, quantitative trait loci (QTL) analyses have revealed numerous loci and candidate genes that might be associated with barley waterlogging tolerance (Zhou, 2011; Broughton *et al.*, 2015; X. Zhang *et al.*, 2016, 2017).

With most breeding efforts focused on shoot biomass and seed yield, it has been suggested that the roots of cereals hold the key to a second green revolution (Gewin, 2010; Maqbool *et al*., 2022). During waterlogging, the root is the first organ to be affected, leading to a drastic reduction of root length and biomass, and decreased root to shoot ratio, impairing the ability of the crop to deal with adverse environmental conditions (Malik *et al*., 2003; Herzog *et al*., 2016). To counteract this, plants must adapt to low oxygen availability in waterlogged conditions through altered metabolism and morphology (Sauter, 2013; Yamauchi *et al*., 2018; J. Pan *et al*., 2021).

Anatomical adaptations to waterlogging include aerenchyma formation (Fagerstedt, 2010), adventitious root development (Jackson and Armstrong, 1999) and a barrier to radial O_2_ loss (Watanabe *et al*., 2013). Aerenchyma, a key trait that promotes waterlogging tolerance (Mustroph, 2018), is a tissue composed of interconnected spaces that enable movement of gases between shoots and roots, consequently, alleviating hypoxia in submerged plant tissues (Jackson and Armstrong, 1999). Faster aerenchyma formation in adventitious (crown) roots under waterlogging stress is associated with increased survival rates in barley (X. Zhang *et al*., 2015), and marker trait associations for aerenchyma formation were previously detected in barley (Broughton *et al*., 2015; X. Zhang *et al*., 2016; Manik *et al*., 2022). Formation of lysigenous aerenchyma involves programmed cell death and is stimulated by ethylene during waterlogging and probably mediated by reactive oxygen species (Sasidharan and Voesenek, 2015). Indeed, respiratory burst oxidases (RBOHs) promoted aerenchyma formation in response to waterlogging stress, and *RBOH* genes were induced by ethylene (Yamauchi *et al*., 2017; Zhang *et al*., 2017; Yamauchi and Nakazono, 2022). Adventitious roots formed in waterlogging stress conditions are rich in aerenchyma and often have an enhanced structural barrier to prevent radial oxygen loss, thereby promoting plant survival (Visser and Voesenek, 2005; Sauter, 2013).

Despite these crucial roles of root system adaptations to survival of waterlogging stress, relatively little is known about the responses of root tissue to waterlogging at the molecular level in barley and, in particular, about the regulation of aerenchyma formation. Recent progress in this area has been observed in terms of characterization of changes in gene expression and proteomic profiling associated with the response to waterlogging in barley roots (Luan *et al*., 2018*a*; Borrego-Benjumea *et al*., 2020), facilitated by the availability of a chromosome-scale assembly of the barley genome (Mascher *et al*., 2017, 2021) and a high-resolution barley transcriptome (Coulter *et al*., 2022). For example, Borrego-Benjumea *et al*. (2020) carried out whole-root-tissue transcriptional profiling under waterlogging stress; subsequently, a larger-scale study examined waterlogging-induced root transcriptomic signatures in 21 barley cultivars that led to isolation of 98 core waterlogging response genes (Miricescu *et al*., 2023). Root transcriptome profiling (Luan *et al*., 2023), and a combination of genome-wide association studies and root transcriptomics (Luan *et al*., 2022), also led to identification of several barley waterlogging-associated genes that were functionally validated in *Arabidopsis thaliana*.

In this study, we explored the transcriptional signatures associated with responses to waterlogging in barley root tissue, focusing on changes in gene expression associated with lysigenous aerenchyma formation. Two barley cultivars that were previously reported to exhibit contrasting waterlogging tolerance, Franklin (sensitive) and Yerong (tolerant) (Li *et al*., 2008; Xue *et al*., 2010; Zhou, 2011; Broughton *et al*., 2015; Luan *et al*., 2018*b*; X. Zhang *et al*., 2015, 2016), were subjected to waterlogging stress. Root aerenchyma formation, adventitious root growth and shoot growth were monitored over the stress duration of 21 days. RNA sequencing was used to characterize changes in gene expression in adventitious root tissue after 24 and 72 h of waterlogging treatment. Two roots were sampled per plant, and notably, we observed a high level of intra-individual variation in the percentage of root aerenchyma in both control and waterlogging stress conditions in both cultivars. We exploited this phenotypic variation to identify genes putatively associated with aerenchyma formation in barley and to infer a gene regulatory network (GRN) underlying development of this trait. Collectively, our data not only further elucidated the transcriptional responses to waterlogging stress in barley root tissue, but also provided specific insights into the genetic regulators underlying formation of aerenchyma, a key anatomical adaptation for waterlogging resilience.

## MATERIALS AND METHODS

### Plant material and growth conditions

Seeds of two barley (*H. vulgare*) cultivars, Franklin and Yerong, were obtained from the Genebank of IPK Gatersleben. Franklin is a two-row winter malting barley accession (Code HOR 15356), whereas Yerong is a six-row spring barley accession (Code BCC 1709) (Li *et al*., 2008). Seeds were surface sterilized in 20 % bleach for 10 min, rinsed at least five times in sterile distilled water and placed on 85 mm Whatman grade 1 filter paper soaked with 5 mL sterile distilled water in 90 mm Petri dishes (six seeds per plate). Petri dishes were sealed with Leukopore tape and placed in the dark for 3 days at 4 °C. Following cold stratification, seeds were placed at 20 °C in the dark for 4 days for germination. Plastic pots (9 cm × 9 cm × 10 cm) were filled to within 1 cm of the top of the pot with autoclaved (40 min, 121 °C) vermiculite as the growth medium (product code VERM, National Agrochemical Distributors Ltd, Dublin, Ireland). Vermiculite was chosen as a semi-structured hydroponics growth substrate to permit the rapid and straightforward collection of clean, undamaged root tissue for subsequent analyses. After 4 days of growth, one seedling was transferred per pot, 1 cm below the surface of vermiculite soaked with 0.25 (quarter strength) Hoagland's solution (pH 5.8) (product code H2395-10L, Sigma-Aldrich, Gillingham, UK). Trays were watered three times weekly from above, with excess Hoagland's solution removed after 30 min. Plant growth conditions were 20 °C (constant temperature), 16 h light–8 h dark photoperiod (85 µmol m^−2^ s^−1^) under fluorescent lamps (product code master TL5 HO 24W840, Philips, Amsterdam, The Netherlands).

### Waterlogging treatment

**Fig. 1.**
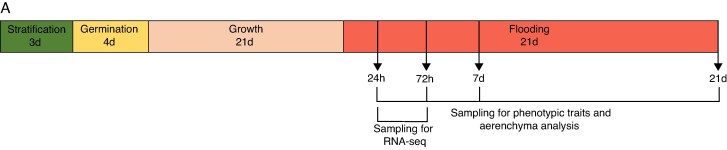
Time line for RNA-seq sampling. Barley plants were grown and sampled according to the presented time line: seeds were cold stratified for 3 days, transferred to 20 °C in the dark for germination for 4 days before planting in vermiculite soaked in 0.25 strength Hoagland's solution. Plants were grown under 16 h light–8 h dark, 20 °C constant-temperature conditions and watered three times a week with 0.25 strength Hoagland's solution from above, with excess removed after 30 min. This was carried out for 21 days, until plants reached the three-leaf stage and were subsequently waterlogged with nutrient solution in plastic bags. Control plants were watered three times a week according to the normal watering schedule. Franklin and Yerong cultivars were sampled at 24 and 72 h for RNA sequencing, and for aerenchyma analysis and phenotypic traits at 1, 3, 7 and 21 days of waterlogging treatment.

Plants were used for waterlogging experiments at 21 days. At this time point, most plants had reached the three-leaf stage (Zadok's stage 13) in the growth conditions described above. To initiate the waterlogging treatment, each pot was placed in a 17 cm × 17 cm plastic bag to prevent drainage, and subsequently placed into an empty 9 cm × 9 cm × 10 cm plastic pot to hold the bag securely in place. The bags were then filled with 0.25 Hoagland's solution (pH 5.8) (product code H2395-10L, Sigma-Aldrich, Gillingham, UK) up to 1 cm above the growth substrate. This level of liquid medium was maintained for the duration of the waterlogging experiment. Control plants were watered as normal (three times per week with 0.25 Hoagland's solution, with the excess removed after 30 min). Plants were waterlogged as detailed in Fig. 1.

### Shoot and root measurements and root sampling

Shoot height (distance from the soil surface to the tip of the tallest leaf in the plant) was initially measured directly prior to initiation of the waterlogging treatment, then at each sampled time point (day 1, 3, 7 and 21). For root phenotyping and sampling, plants were removed from the pots and the root system gently washed with water to remove vermiculite, followed by recording the number of adventitious (crown roots) roots for day 1, 3 and 7. The lengths of all individual adventitious roots were also measured and added to calculate the total length of adventitious roots for each plant. Owing to high numbers of roots and difficulty in separating highly interwoven roots without causing mechanical damage, these measurements were not taken after 21 days of treatment. Furthermore, for quantification of aerenchyma (day 1, 3, 7 and 21) and transcriptomic profiling (day 1 and 3), two adventitious roots were sampled per plant to gain insights into intra-individual variation associated with aerenchyma formation. Sampled roots were ≥6 cm long, and it was attempted to select roots of average length that were not damaged by removal of the growth substrate. For each sampled root, the 2-cm-long section from 1–3 cm away from the root tip was flash frozen in liquid nitrogen and stored at −80 °C until RNA extraction. Additionally, the 2-cm-long root section from 3–5 cm away from the root tip was collected and stored in water for quantification of aerenchyma. Three independent repeats of the experiment were carried out. For every cultivar–treatment combination, *n* = 4 plants were sampled at days 1 and 3 of waterlogging (total of 12 biological replicates across three experimental repeats), with two roots sampled per plant. Owing to limited growth space available, the number of replicates per experiment was reduced to two or three for day 7 of waterlogging and three or four for day 21 of waterlogging. As a result, the total number of plants (biological replicates) examined across three experimental repeats for each cultivar–treatment combination were as follows: day 7: Yerong control, 6; Yerong waterlogged, 7; Franklin control, 5 and Franklin waterlogged, 8; day 21: Franklin control, 9; Franklin waterlogged, 9; Yerong control, 10; and Yerong waterlogged, 9. For one of the control Yerong plants at day 21 of waterlogging, only one sufficiently long (>6 cm) adventitious root was available for sampling; for all other plants, two roots were collected successfully.

SPSS v.27 (IBM Corp, 2020) was used for statistical analyses of phenotypic traits. Analyses were carried out using two-way ANOVAs (*P*-value = 0.05), with Bonferroni correction for multiple testing using estimated marginal means.

### Hand-sectioning of adventitious roots and quantification of aerenchyma

Collected root segments were placed in individual vertically positioned moulds constructed from 2 mL syringes with the tip cut off, and embedded in 5 % (w/v) liquid agar (product code A1296, Sigma-Aldrich, Gillingham, UK) (Supplementary Data Fig. S1). The embedded roots were sliced manually into thin sections (∼0.25 mm thick) using a double-edged razor blade (Solimo, Washingon, USA). The sections were kept in water, until imaging on microscope slides using a Leica DM500 light microscope connected to a Leica MC170 HD camera (×100 magnification).

For quantification of aerenchyma, images were analysed with ImageJ v.1.50i (Schneider *et al*., 2012) to calculate the overall cross-sectional area of the root, number of aerenchyma lacunae and area of aerenchyma for each sample. The percentage of aerenchyma was calculated based on the area of aerenchyma relative to the total cross-sectional area of the root.

SPSS v.27 (IBM Corp, 2020) was used for statistical analyses of aerenchyma measurements. Briefly, analyses were carried out using a two-way ANOVA (*P*-value = 0.05), with Bonferroni correction for multiple testing using estimated marginal means for general aerenchyma percentages with log_10_-transformed values. A two-way repeated-measures ANOVA (*P*-value = 0.05) was carried out for intra-individual (root to root) aerenchyma percentage analysis.

### Root homogenization, sample pooling, RNA extraction and sequencing

Frozen root tissue was homogenized manually in liquid nitrogen in 1.5 mL Eppendorf tubes using microtube pestles and 0.1 mm glass beads (product code 11079-101, Biospec Products, Inc., Oklahoma, USA). The pooling of individual samples of homogenized root sections prior to RNA extraction was informed by the previously performed aerenchyma quantification that revealed high intra-individual (root to root) variation in this trait. Briefly, for two adventitious roots collected from each plant, the root with lower aerenchyma percentage was categorized as L_AE and the root with higher aerenchyma percentage as H_AE (i.e. for each pair of roots taken from the same plant, one was classified as higher aerenchyma percentage and one classified as lower aerenchyma percentage). For each experimental repeat, for each time point (1 and 3 days), condition (control and waterlogging) and cultivar (Yerong and Franklin), the four H_AE homogenized root samples were pooled together, with the same done for four L_AE homogenized root samples. Subsequently, total RNA was extracted using the Qiagen RNeasy Plant Mini Kit (product code 74904, Qiagen, MD, USA) with an on-column DNase digestion step (RNase-Free DNase Set, product code 79254, Qiagen, MD, USA). The obtained RNA was quantified using an ND1000 Nanodrop (Thermo Scientific, MA, USA) and its integrity determined using an Agilent 2100 Bioanalyzer (Agilent). Library preparation and mRNA sequencing (NovaSeq PE150, 6G raw data per sample, poly-A selection) was carried out by Novogene (Cambridge, UK). In total, 48 RNA sequencing (RNA-seq) datasets were generated: three experimental repeats with two cultivars × two time points × two treatments × two aerenchyma categories (H_AE and L_AE) (Supplementary Data Table S1).

### Differential gene expression analysis

The differential gene expression (DGE) analysis was performed using Galaxy's graphical user interface for quality checking and trimming (https://usegalaxy.eu/; Afgan *et al*., 2018), through the command line for mapping and expression quantification and, finally, through R for differential gene expression analysis. Initially, the quality of raw reads was assessed with FastQC (v.0.11.9) (Andrews, 2010) using default settings. The sliding-window function in Trimmomatic (v.0.38.1) (Bolger *et al.*, 2014) was used to trim windows of four bases below an average Phred score of <20 (99 % accuracy of base call) and, in addition, the ILLUMINACLIP function was used to remove custom adapter sequences. Trimmed reads were mapped to the most comprehensive and resolved reference transcriptome available for barley, BaRTv2.18 (https://ics.hutton.ac.uk/barleyrtd/bart_v2_18.html; Coulter *et al*., 2022), through the command line with Salmon (v.0.12.0) (Patro *et al*., 2017), using --validateMappings and --gcBias parameters. Raw expression counts of the transcripts based on BaTRv2 gene-transcript annotation were aggregated according to the gene-level counts using the R package *tximport* (Soneson *et al*., 2016). Subsequently, DGE analysis was carried out in RStudio (v.2023.06.2) (RStudio Team, 2020) through R (R v.4.2.2) (R Core Team, 2022) using the package *DESeq2* (Love *et al*., 2014).

Two different types of DGE analyses were performed. First, to identify genes that might be associated with aerenchyma formation, the H_AE versus L_AE comparisons were performed for each time point, condition (waterlogging and control) and cultivar. Second, for characterization of the effect of waterlogging treatment itself on gene expression (Waterlogged, WL, versus control, CRT, comparisons), the H_AE and L_AE datasets for each time point, condition and cultivar were combined for each experiment using the DESeq2 function *collapseReplicates* (Supplementary Data Table S1). In all analyses, the genes with <10 counts were removed, and the Benjamini–Hochberg (Benjamini and Hochberg, 1995) adjusted *P*-value cut-off of 0.05 was applied. To generate UpSet plots, the package *UpSetR* was used (Conway *et al*., 2017). Finally, the R package *EnhancedVolcano* (Blighe *et al*., 2018), with log_2_fold change cut-off set to two and *P*-value cut-off set to 1 × 10^−6^, was used for WL versus CRT comparisons, and log_2_fold change cut-off set to 0.5 and *P*-value cut-off set to 1 × 10^−3^ was used for H_AE versus L_AE to generate volcano plots (Supplementary Data Figs S2–S4).

### Enrichment analyses

Although BaRTv2 is a highly resolved barley reference transcriptome recommended to ensure accurate transcript-specific RNA-seq quantification (Coulter *et al*., 2022), BaRTv2-based annotations are not compatible with the available gene enrichment tools, typically based on previous barley reference assemblies, such as the MorexV3 genome (Mascher *et al*., 2021). Therefore, the BaRTv2 annotations of genes isolated by DGE analyses were initially converted to corresponding MorexV3 (Mascher *et al*., 2021) genome gene names using a file obtained from Dr Linda Milne (Supplementary Data Table S2). This conversion of gene identities (IDs) currently provides corresponding MorexV3 IDs for only ∼63 % of BaRTv2 IDs, which corresponded to 38 of 81 aerenchyma-associated differentially expressed genes (DEGs). This is an issue commonly observed during the conversion process between gene formats. The gene enrichment analyses for identified: (1) waterlogging-responsive; and (2) aerenchyma-associated genes, were subsequently carried out on MorexV3 gene IDs using ShinyGO v.0.75c (Ge *et al*., 2020). ShinyGO output was set to 30 gene ontology (GO) terms with a false discovery rate (FDR) cut-off 0.05. Additionally, given that the key focus of this study was identification of mechanisms underlying the formation of root aerenchyma and owing loss of data in conversion from BaRTv2 to MorexV3 IDs, another GO enrichment tool in PlantRegMap (2019-10-11) (Jin *et al*., 2015, 2017; Tian *et al*., 2020) was also used. *Arabidopsis thaliana* homologues were first identified using the PlantRegMap ID mapping tool by inputting BaRTv2 protein FASTA sequences of the 81 aerenchyma-associated genes, resulting in identification of 33 homologous *A. thaliana* genes. These 33 *A. thaliana* homologues were used for the PlantRegMap GO term enrichment (*P-*value cut-off 0.01). Additionally, the motif-based transcription factor enrichment function of PlantRegMap was used (*P*-value cut-off 0.01) to identify the putative upstream transcriptional regulators of this gene list.

### Gene regulatory network construction

The GRN underlying aerenchyma formation was created using the GeneMANIA v.3.5.2 (Warde-Farley *et al*., 2010) plug-in in Cytoscape v.3.9.1 (Shannon *et al*., 2003). *Arabidopsis thaliana* homologues identified for isolated aerenchyma-associated BaRTv2 barley genes were used as an input, because the GeneMANIA tool is not compatible with the barley gene annotations. Addition of ≤20 predicted nodes was permitted. The GRN was clustered using clusterMaker2 v.2.3.4 (Morris *et al*., 2011) with MCL (Markov clustering algorithm) clustering enabled and network granularity set to four. Node size was set to correspond to the degree of connectivity of nodes. Hub genes were identified based on nodes that were most connected in the network.

### Cross-study comparison

A comparison between results obtained here and the universal barley 98 hypoxia-responsive genes (MorexV3 annotation) recently reported by Miricescu *et al*. (2023) was performed. The available BaRTv2 gene IDs were retrieved for 98 hypoxia-responsive genes from Miricescu *et al*. (2023), and *GeneOverlap* package in R (Shen, 2023) was used to identify the genes that were also transcriptionally responsive to waterlogging in this study.

## RESULTS

### Root system adaptations mediating waterlogging tolerance in barley

**Fig. 2.**
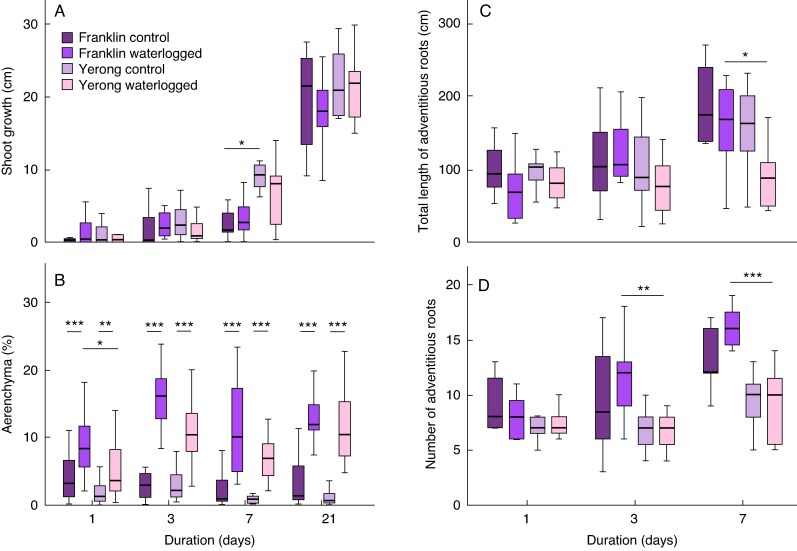
Response to waterlogging treatment observed in barley cultivars Franklin and Yerong. The waterlogging treatment was initiated at the three-leaf stage. (A) Shoot growth was determined by measuring plant height prior to start of the treatment and after 1, 3, 7 and 21 days of waterlogging treatment. (B) Aerenchyma percentage in adventitious roots. Roots (two per plant) were sampled 3–5 cm from the root tip, and log_10_-transformed values were used for statistical analyses. (C) Mean total length of adventitious roots per plant. (D) Mean number of adventitious roots for each plant. Bars represent mean, error bars represent 95 % confidence interval. ***P ≤ 0.001, **P ≤ 0.01 and *P ≤ 0.05 after two-way ANOVA (*P*-value = 0.05) with Bonferroni correction for multiple testing using estimated marginal means in SPSS. Duration corresponds to the duration of treatment. Experiments were repeated three times, with *n* = 4 plants sampled per cultivar–treatment combination at days 1 and 3 of waterlogging, *n* = 2–3 plants at day 7, and *n* = 3–4 at day 21.

Waterlogging did not affect the shoot growth during the 21-day treatment in Franklin and Yerong plants (Fig. 2A). Although the shoot growth rate is only a limited proxy for waterlogging tolerance, this could suggest that responses effectively mitigating waterlogging stress were induced in both cultivars. Accordingly, both Franklin and Yerong demonstrated a significant increase in levels of the waterlogging adaptive trait, aerenchyma, as early as after 1 day of treatment (Fig. 2B). A significant difference in aerenchyma percentage between the two cultivars was observed only between plants waterlogged for 1 day (Franklin, 9.9 % versus Yerong, 5.95 %, *P* = 0.017), potentially indicating a slightly faster aerenchyma formation in Franklin. Waterlogging treatment did not have a significant effect on the number and total length of adventitious roots (Fig. 2C, D). However, the number of adventitious roots in waterlogged plants was higher in Franklin at day 3 and 7, and the total root length was also higher at day 7 of waterlogging for this cultivar. This suggests that although there were no differences in levels of aerenchyma between waterlogged Franklin and Yerong at these time points, the total volume of aerenchyma tissue available for gas exchange might be higher in Franklin.

### RNA sampling strategy to characterize waterlogging response in barley roots and isolate novel candidates for aerenchyma-associated genes

**Fig. 3.**
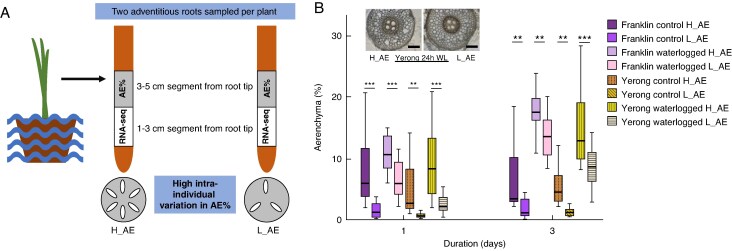
Observation of intra-individual variation in aerenchyma formation in adventitious roots. Following 1 and 3 days of treatment, root systems were washed in water, and two roots were sampled from each plant for RNA sequencing (segment 1–3 cm from the root tip) and aerenchyma percentage analysis (segment 3–5 cm from the root tip). For aerenchyma analysis, roots were embedded in liquid agar prior to hand-sectioning thin cross-sections of root tissue and imaging using a light microscope at ×100 magnification. Percentage aerenchyma was quantified for each root using ImageJ software. From each plant, both roots were compared and categorized as high (H_AE) or low (L_AE) aerenchyma-forming roots relative to one another, which led to the detection of high intra-individual aerenchyma percentage in roots sampled from the same plant (A, B). The graph indicates intra-individual variation in aerenchyma percentage of roots from the same plant, and the images represent an example of intra-individual variation in % AE between two roots from the same waterlogged plant (cultivar Yerong; scale bar: 200 µm). Duration corresponds to the duration of treatment. ***P ≤ 0.001 and **P ≤ 0.01 based on two-way repeated-measures ANOVA (*P*-value = 0.05) with Bonferroni correction for multiple testing using estimated marginal means; error bars display 95 % confidence interval. Experiments were repeated three times; each time, *n* = 4 plants were analysed for each cultivar, treatment, time point and aerenchyma group.

Aerenchyma formation was induced in both Franklin and Yerong after day 1 of waterlogging treatment, and its formation was enhanced further after day 3 of treatment, suggesting that this sampling window is suitable for the capture of changes in gene expression associated with aerenchyma formation. Furthermore, although in general there is a relatively limited number of datasets representing barley root response to waterlogging, the time points between 1 and 3 days were also used by several recently published studies exploring the response to waterlogging (Borrego-Benjumea *et al*., 2020; Luan *et al*., 2023). Using the same time points, therefore, might facilitate comparisons between the gene expression response to waterlogging observed across several datasets, different experimental set-ups and cultivars. When analysing the root aerenchyma percentage, we also noted the high variability in aerenchyma percentage in the sampled adventitious roots observed for both cultivars (Fig. 2B). Notably, the intra-individual variability in aerenchyma formation was substantial, thus offering an opportunity to isolate genes associated with aerenchyma formation by comparing gene expression profiles of root samples that differ in aerenchyma percentage within each condition and cultivar. We tested the extent of intra-individual variability in aerenchyma formation by measuring the aerenchyma percentage in the two adventitious roots collected from each plant; subsequently, the root with lower aerenchyma percentage for each plant was labelled as L_AE and the root with higher aerenchyma percentage as H_AE (Fig. 3A). The difference in aerenchyma percentage between L_AE and H_AE roots was significant at time points for which tissue was sampled for RNA sequencing (day 1 and 3), in both control and waterlogging conditions, in both cultivars (Fig. 3B). To exploit the potential of this sizeable intra-individual variability in aerenchyma formation for identification of aerenchyma-associated genes, during each independent experimental repeat, the RNA from pooled H_AE and pooled L_AE roots collected was sequenced separately for each time point (1 and 3 days), condition (control and waterlogging) and cultivar (Yerong and Franklin). Consequently, combining the H_AE and L_AE datasets facilitated determination of the response to waterlogging stress for each time point and cultivar, whereas comparisons of matched H_AE and L_AE datasets enabled high-resolution isolation of genes responsible for differences in aerenchyma formation.

### Transcriptional response to waterlogging stress in barley roots: pathways related to redox state management, metabolism and stress responses

**Fig. 4.**
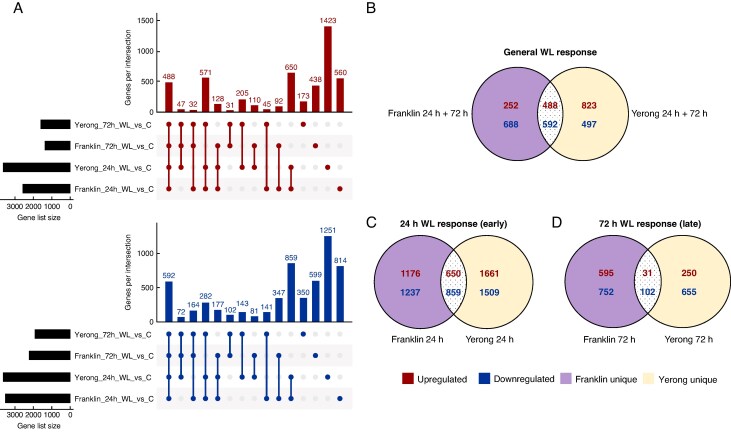
Transcriptional response to waterlogging stress in barley roots. (A) UpSet plots representing the overlapping DEGs upregulated (red) and downregulated (blue) in response to waterlogging (WL) at 24 and 72 h treatment in comparison to the respective control in each cultivar. Gene list size represents the number of DEGs for each comparison, and genes per intersection corresponds to the number of shared DEGs among the comparisons marked as connected dots below. The single dots represent DEGs unique to one comparison. Each comparison represents DEGs under waterlogging treatment with control as the baseline level. (B) Venn diagram representing the general waterlogging responses. General waterlogging response genes are those commonly differentially regulated at point time points and in both cultivars. (C, D) The number of DEGs unique to the 24 h (C) and 72 h (D) time points are shown in respective Venn diagrams indicating DEGs common to both cultivars and uniquely differentially expressed. Upregulated genes are shown in red and downregulated genes in blue. Franklin unique expressed genes are highlighted in purple areas; Yerong unique genes are shown in yellow areas.

Differential gene expression analysis was carried out to characterize the transcriptional response of barley roots to 24 and 72 h of waterlogging stress, using pooled H_AE and L_AE datasets for each time point, condition and cultivar. This revealed extensive changes in gene expression induced in both cultivars (Fig. 4A; Supplementary Data Table S3A). Furthermore, we isolated cultivar-specific and core DEGs, regulated in the same direction at both time points (‘general waterlogging response’; Fig. 4B) or unique to only 24 h (early waterlogging response; Fig. 4C) or 72 h (late waterlogging response; Fig. 4D) time points. This revealed a greater overlap between the cultivars for the general (32 % overlap) and early (21 % overlap) transcriptional response to waterlogging, compared with transcriptional changes unique to late (72 h) time points, where only ∼6 % of DEGs were differentially regulated in both Yerong and Franklin.

Subsequently, we investigated whether differences in basal gene expression between the cultivars could explain the sizeable differences in waterlogging responses between Franklin and Yerong. However, although there were large differences in basal (control) gene expression between Franklin and Yerong at both 24 and 72 h, they provided putative explanation for only between 9 and 26 % of cultivar-specific differences in gene expression in response to waterlogging (Supplementary Data Fig. S5). The transcriptional signature induced by waterlogging stress in this study was also compared with the list of core waterlogging-response genes recently identified by Miricescu *et al*. (2023), revealing a significant overlap and strong association between the response observed for both cultivars and at both time points (Supplementary Data Table S4A), with the same direction of regulation (Supplementary Data Table S4B). Collectively, the generated data underscore that both core and cultivar-specific responses to waterlogging stress exist and need to be dissected further to elucidate transcriptional signatures related to waterlogging tolerance and sensitivity.

**Fig. 5.**
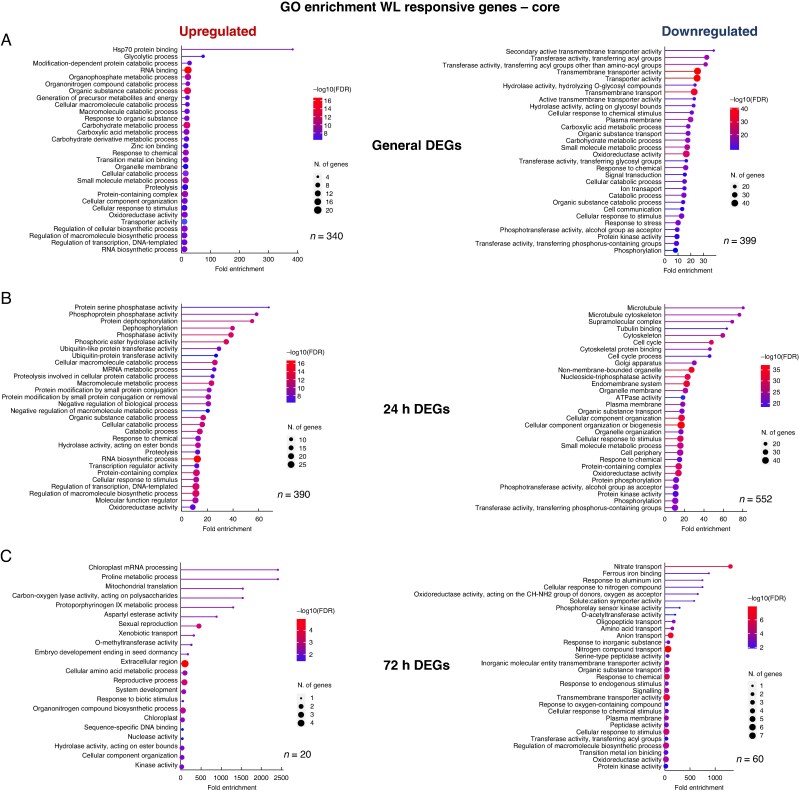
Gene ontology (GO) enrichment of WL response genes. Upregulated and downregulated DEGs associated with core (both cultivars), for general (both time points) (A), 24 h unique (B) and 72 h unique (C) time points were identified following DESeq2 analysis. BaRTv2 gene names were converted to MorexV3 gene names (represented by *n* for each GO analysis) and subsequently input into ShinyGO v.0.75c. ShinyGO output was set to 30 GO terms and FDR cut-off as 0.05. The lollipop graph shows fold enrichment on the *x*-axis with the size of dot representing number of genes associated with a particular GO term. Colour of line and dot represents −log_10_(FDR).

To explore further the core and cultivar-specific responses to waterlogging stress and obtain insights into relevant biological pathways, GO enrichment analysis was carried out on the isolated DEGs using ShinyGO (Ge *et al*., 2020) (Fig. 5; Supplementary Data Figs S6 and S7).

As anticipated, the general (both time points) core (both cultivars) response to waterlogging resulted in enriched pathways involved in metabolism, stress mitigation and redox state, pathways that are central components of stress responses in plants (Armstrong and Drew, 2002; Yamauchi *et al*., 2018; J. Pan *et al*., 2021; Teoh *et al*., 2022). For example, the upregulated general core genes were enriched in glycolytic processes, catabolic processes and oxidoreductase activity, in addition to pathways associated with metabolism modulation and response to stress/stimuli (e.g. ‘carbohydrate metabolic process’, ‘small molecule metabolic process’, ‘response to chemical’ and ‘Hsp70 protein binding’) (Fig. 5). The downregulated general core DEGs were enriched for oxidoreductase activity, catabolic processes, response to stimuli and transferase and transporter activity. This suggests that the general waterlogging response, independent of cultivar or stress duration, is composed of mitigating stress through modulation of metabolism, activating stress responses and managing the redox state to promote plant homeostasis.

The early (24 h) core waterlogging response up- and downregulated DEGs reiterated the importance of redox regulation in waterlogging response (‘oxidoreductase activity’) but also highlighted phosphorylation state-associated terms (e.g. ‘protein serine phosphatase activity’, ‘phosphoprotein phosphatase activity’, ‘dephosphorylation’ and ‘phosphorylation’) and stimuli/stress response-associated terms (e.g. ‘response to chemical’ and ‘cellular response to stimulus’), suggesting that modulation of phosphorylation state is strongly associated with an early stress response in barley. The early core waterlogging upregulated DEGs also showed enrichment in additional posttranslational modification/protein modification pathways, including ubiquitination (e.g. ‘ubiquitin-like protein transferase activity’, ‘ubiquitin protein transferase activity’, ‘protein modification by small protein conjugation’ and ‘protein modification by small protein conjugation or removal’), further underscoring the importance of posttranslational modifications in modulating the waterlogging response in barley. Interestingly, early (24 h) core waterlogging downregulated DEGs were enriched in terms including ‘cell cycle’ and ‘cell cycle process’ that might suggest early arrest of the cell cycle during waterlogging stress. However, given that bulk root tissue was used for RNA-seq, spatial transcriptomic profiling approaches should be used in the future to ascertain whether this effect is highly localized and/or limited to particular cell types.

The later (72 h) specific core response upregulated DEGs highlighted GO terms related to hydrolase, transferase, lyase and esterase enzyme activities (e.g. ‘carbon-oxygen lyase activity, acting on polysaccharides’, ‘aspartyl esterase activity’, ‘O-methyltransferase activity’ and ‘hydrolase activity, acting on ester bonds’) that might allude to enzymatic modulation of cell wall components during waterlogging. Some enrichment of phosphorylation state-related pathways was also observed for later (72 h) up- and downregulated core responses (‘kinase activity’ and ‘protein kinase activity’), suggesting that this posttranslational modification plays a role in regulating waterlogging responses across time points.

Furthermore, gene enrichment analysis of cultivar-specific, core, early (24 h) and later (72 h) responses to waterlogging suggested that at the pathway level the gene expression changes observed in Yerong and Franklin are generally more similar than at gene list level (Supplementary Data Figs S6 and S7), highlighting terms associated with oxidoreductase activity, catabolic/metabolic processes, cell wall modification and responses to stress/stimuli.

### Isolation of aerenchyma-associated genes and constructing gene regulatory networks underlying aerenchyma formation in barley

**Fig. 6.**
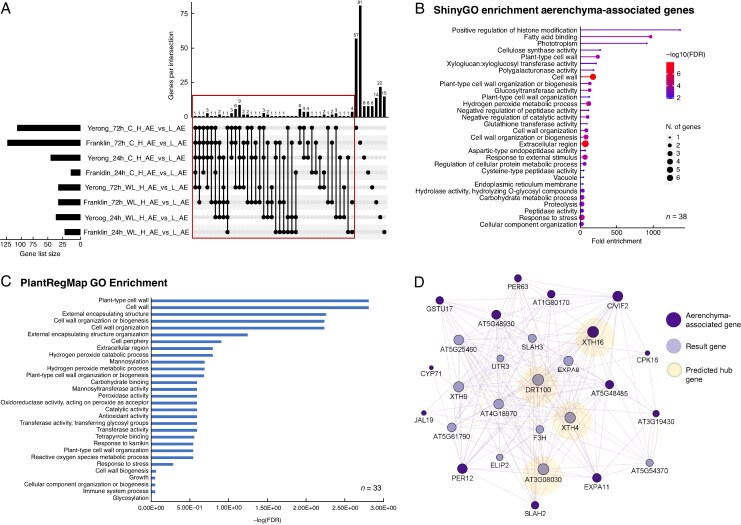
Harnessing intra-individual variability in aerenchyma formation to identify aerenchyma-associated transcriptomic response in Franklin and Yerong barley cultivars. (A) Differential gene expression analysis was carried out for high percentage aerenchyma (H_AE) versus low percentage aerenchyma (L_AE) comparisons in both treatments (waterlogging or control), both cultivars (Franklin or Yerong) and both time points (24 h or 72 h), with results summarised in the UpSet plot. Gene list size represents the number of DEGs for each sample for each comparison, and genes per intersection represents the number of overlapping DEGs between specific comparisons as indicated by the connected dots below. Single dots represent unique DEGs to one comparison. Each comparison represents DEGs in high percentage aerenchyma forming sample with low percentage aerenchyma sample as the baseline level. Red box indicates aerenchyma-associated DEGs present in at least two of eight of the high versus low percentage aerenchyma comparison groups (81 DEGs total). (B, C) GO enrichment of aerenchyma-associated DEGs. Aerenchyma-associated DEGs (BaRTv2 gene format) were converted to MorexV3 gene name where possible, with *n* indicating the number of genes converted to MorexV3 format. (B) MorexV3 gene names were subsequently input ShinyGO v.0.75c for GO enrichment. Output was set to 30 GO terms and FDR cut-off as 0.05. (C) Aerenchyma-associated DEGs were input as BaRTv2 protein FASTA sequences into the ID mapping tool (BLASTp) in PlantRegMap to obtain *Arabidopsis thaliana* IDs; *n* represents number of DEGs which had a corresponding *A. thaliana* ID. The *A. thaliana* IDs were input into the GO term enrichment tool in PlantRegMap, with *P*-value cut-off of 0.01. (D) Clustered gene regulatory network (GRN) of aerenchyma-associated genes with predicted hub genes. Aerenchyma-associated genes (dark purple) with corresponding *Arabidopsis* gene IDs (*n* = 33) were input into Cytoscape using the GeneMANIA v.3.5.2 plugin, where 20 resulting (light purple) nodes were inserted based predicted gene interactions. Node size corresponds to the degree of connectivity of nodes, with larger nodes being more connected. Hub genes are identified by nodes with the greatest connectivity and circled in green. The GeneMANIA network was clustered using the Cytoscape plugin clusterMaker2 v.2.3.4 with MCL clustering, granularity parameter set to 4. Interaction links in purple are predicted based on gene co-expression, in blue are co-localized genes, and yellow are genes with shared protein domains. The edge thickness in the GRN corresponds to normalized maximum weight.

Differential gene expression analysis was carried out to compare the transcriptional profile of H_AE and L_AE samples within the same treatment (same time point, condition and cultivar) (Fig. 6A). The 81 DEGs (annotation based on that BaRTv2 transcriptome) that were present in at least two of H_AE versus L_AE comparisons were considered putatively to be associated with aerenchyma formation and used for further analyses (Supplementary Data Table S3B). Among these aerenchyma responsive genes were three peroxidases, two kinases (one calcium dependent), two glycosyltransferases and a proteolytic enzyme, bromelain. Moreover, 19 of the 81 isolated DEGs were differentially regulated by waterlogging stress for at least one time point, for at least one cultivar (Supplementary Data Table S3C). Some of the most interesting candidates from this aerenchyma-associated, waterlogging-induced gene list include peroxidases, which have been implicated in positive regulation of programmed cell death responses in plants (Graças *et al*., 2020; J. Zhang *et al*., 2022), and an S-type anion channel, SLAH3 (encoded by *BaRT2v18chr1HG047880*), that was previously associated with plant immune responses (Y. Liu *et al*., 2019).

Furthermore, to investigate pathways that might be involved in the regulation of aerenchyma formation in barley roots, GO enrichment analysis was performed using ShinyGO (Fig. 6B) and PlantRegMap (Fig. 6C) tools. These GO term enrichment analyses (Fig. 6B, C) highlighted pathways associated with cell wall organization/modification (e.g. ‘plant-type cell wall organization or biogenesis’, ‘plant-type cell wall’, ‘cell wall organization’, ‘cell wall’, ‘cell wall organization or biogenesis’, ‘xyloglucan:xyloglucosyl transferase activity’ and ‘cellulose synthase activity’), stress responses (e.g. ‘response to external stimulus’, ‘response to stress’ and ‘response to karrikin’), redox state management (‘glutathione transferase activity’, ‘hydrogen peroxide metabolic process’, ‘peroxidase activity’, ‘antioxidant activity’, ‘oxidoreductase activity, acting on peroxide as acceptor’, ‘hydrogen peroxide metabolic process’, ‘hydrogen peroxide catabolic process’ and ‘reactive oxygen species metabolic process’) and regulation of plant immunity (‘immune system process’). This provided validation of the isolated gene list of aerenchyma-associated genes, considering that lysigenous aerenchyma formation involves programmed cell death of root cortical cells and is a process associated with cell wall modifications (Saab and Sachs, 1996; Gunawardena *et al*., 2001*b*), management of reactive oxygen species signalling (Yamauchi *et al*., 2017; Ni *et al*., 2019; R. Pan *et al*., 2021, 2022) and modulation of immune signalling pathways (Mühlenbock *et al*., 2007; Karpinski *et al*., 2013). Interestingly, several enriched terms also suggested less explored pathways that might play a role in aerenchyma formation, or programmed cell death in general, and should be investigated further in this context, including regulation of histone modification and response to karrikins.

Subsequently, to elucidate further the genetic regulation of aerenchyma formation and isolate genes likely to play a central role in this process, we constructed a GRN using Cytoscape (Shannon *et al*., 2003) and the GeneMANIA (Warde-Farley *et al*., 2010) plugin with 33 identified *A. thaliana* homologues of aerenchyma-associated genes as an input (Fig. 6C). The constructed GRN revealed a cluster of highly connected nodes (Fig. 6D). The four most connected (≥20 connections) genes from this cluster were classified as hub genes, because they can play a central regulatory role in the constructed network and therefore warrant further investigation of their role in the context of aerenchyma formation. These hub genes were *DRT100* (DNA-DAMAGE REPAIR/TOLERATION 100), which plays a role in DNA damage repair and tolerance (Ghosh *et al*., 2013), *XTH16* (XYLOGLUCAN ENDOTRANSGLUCOSYLASE/HYDROLASE 16), *AT3G08030* (encoding a cell wall protein DUF642) and *XTH4* (XYLOGLUCAN ENDOTRANSGLUCOSYLASE/HYDROLASE 4). Finally, we identified 39 putative upstream transcriptional regulators with significantly overrepresented targets within the aerenchyma-associated gene list (Supplementary Data Table S5). Notably, many of the identified transcription factors were previously linked to stress responses (including water stress), plant immunity and programmed cell death, with most of them (30) belonging to the ethylene response factor (ERF) family. Ethylene is a crucial signal transducer that plays a central role in plant adaptation responses to low oxygen, including induction of aerenchyma (He *et al*., 1996; Gunawardena *et al*., 2001*a*; Yukiyoshi and Karahara, 2014; Ni *et al*., 2019), in addition to adventitious root formation (Visser *et al*., 1996; Negi *et al*., 2010; Qi *et al*., 2019), and numerous ERFs were previously reported to mediate these responses (Hattori *et al*., 2009; Licausi *et al*., 2010; Kitomi *et al*., 2011; Trupiano *et al*., 2013; Yang, 2014).

## DISCUSSION

Barley is a waterlogging-susceptible cereal crop. Waterlogging-induced yield losses in this species vary from 10 to 50 %, depending on the stress duration, plant developmental stage, soil type and temperature (Setter *et al*., 1999; Setter and Waters, 2003; Pang *et al*., 2004). Increased understanding of the genetic regulation underlying waterlogging-tolerance mechanisms in barley is therefore urgently required to inform breeding programmes and to support development of climate-proofed, more waterlogging-tolerant barley cultivars. The root system, as the first line of defence against soil waterlogging, undergoes metabolic and anatomical adaptations supporting survival of the whole plant and should therefore be studied as a priority when early waterlogging stress responses are considered (Herzog *et al*., 2016; Langan *et al*., 2022). However, relatively limited resources are currently available in terms of transcriptomic (Borrego-Benjumea *et al*., 2020; Luan *et al*., 2023), proteomic or metabolomic datasets (Luan *et al*., 2018*a*, 2022; Andrzejczak *et al*., 2020) characterizing responses of barley root tissue to waterlogging stress or hypoxia. Here, we used a combination of plant phenotyping and transcriptomics (RNA-seq) to improve our understanding of waterlogging responses in barley root tissue.

The focus of the study was the identification of new signalling pathways and genes modulating formation of root aerenchyma, a key anatomical adaptation for waterlogging tolerance (Mustroph, 2018). We achieved this by carrying out quantification of aerenchyma in the roots that were subsequently used for transcriptional profiling. This not only uncovered a high intra-individual variation in root aerenchyma levels in both control and waterlogged conditions, but also facilitated comparisons of gene expression between roots with high and low levels of aerenchyma, leading to identification of putative aerenchyma-associated genes and inference of GRN for this trait. The two barley cultivars used were selected owing to previous reports on their contrasting tolerance to waterlogging, with Yerong generally being reported to be more waterlogging tolerant and demonstrating more extensive aerenchyma formation compared with Franklin (Li *et al*., 2008; Xue *et al*., 2010; Zhou, 2011; Broughton *et al*., 2015; X. Zhang *et al*., 2015, 2016; Luan *et al*., 2018*b*). However, in this study, both cultivars showed no reduction in shoot growth, which can potentially be explained by the observed fast induction of aerenchyma in root tissue, indicating activation of stress adaptation responses. It is also possible that the duration of this study was not sufficient to observe a significant impact of waterlogging stress on the shoot growth, which might become apparent only after a longer stress period or during subsequent stress recovery, as previously reported (Miricescu *et al*., 2021). The percentage of root aerenchyma increased after 24 h of waterlogging in both cultivars, in line with other studies reporting development of this trait over a similar stress period (X. Zhang *et al*., 2015, 2016; Manik *et al*., 2022; Teoh *et al*., 2022). However, in the present study the aerenchyma levels appeared similar in both cultivars over the investigated period, apart from a small but significant difference of 3.95 % in percentage aerenchyma between Franklin and Yerong after 24 h of waterlogging, potentially arising from slightly faster formation of aerenchyma in Franklin roots. Furthermore, despite previous reports on waterlogging promoting formation of adventitious roots (Visser *et al*., 1996; Chen *et al*., 2002; X. Qi *et al*., 2019; Koramutla *et al*., 2022), we did not observe differences in the total length and number of adventitious roots over the investigated time period as a result of the waterlogging treatment applied. However, in waterlogging conditions, the number of adventitious roots (at day 3 and 7) and total root length (at day 7) were lower in Yerong compared with Franklin. These observations contrast with previous results for these cultivars, with, for example, Luan *et al*. (2018*b*) reporting an increase in both total adventitious root length and number in waterlogged compared with control treatments in Yerong, but not in Franklin. These observations underscore that the response of cultivars to waterlogging can depend on the exact experimental set-up used (type of growth substrate, time/duration of treatment application, temperature, etc.), often making it difficult to cross-compare the results (Miricescu *et al*., 2021). In the present study, plants were grown in vermiculite, to facilitate collection of clean root tissue for RNA extraction and to enable replicability of the experimental set-up by other studies. However, the use of vermiculite would also result in less compacted growth substrate in comparison to studies that use peat mix (e.g. Luan *et al*., 2018*b*), potentially alleviating the impact of applied waterlogging stress, because the link between soil compaction and the impact of waterlogging has been suggested previously (Grzesiak *et al*., 2016; X. Wu *et al*., 2018). Neither cultivar tested here showed a significant reduction in shoot growth under waterlogging, possibly thanks to induced stress adaptations.

### Core and cultivar-specific transcriptional response to waterlogging in barley roots

Analysis of RNA-seq datasets, generated from collected root tissue, facilitated identification of core and cultivar-specific genes differentially expressed in response to 24 and 72 h of waterlogging. The transcriptomic analyses were performed using a highly resolved barley reference transcriptome that allows accurate transcript-specific RNA-seq quantification (BaRTv2) (Coulter *et al*., 2022). It needs to be highlighted that downstream analyses were challenging because of differences in gene identifiers between transcriptomic (BaRTv2) and genomic (MorexV3) resources for barley; for example, the gene enrichment analysis tool (ShinyGO) used in the present study is compatible only with the MorexV3 gene IDs, requiring conversion of BaRTv2 identifiers to MorexV3, which, in our case, reduced the number of DEGs by ∼37 %.

**Fig. 7.**
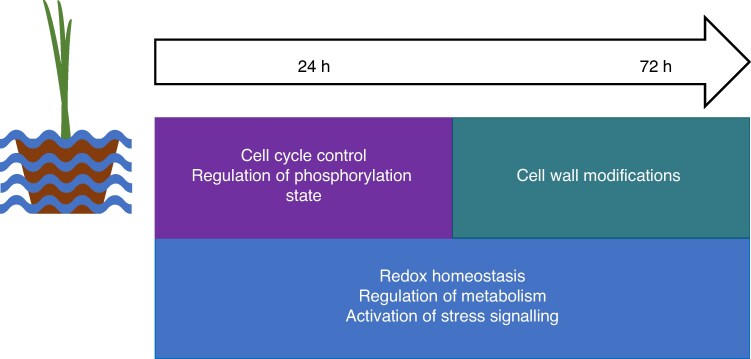
Summary of transcriptional changes in barley roots under waterlogging stress.

The changes in gene expression observed at both time points, in both cultivars, highlighted GO terms associated with redox state management, metabolic adaptations and stress responses, suggesting that these processes play a key role in the waterlogging response in barley (Fig. 7). These GO terms have previously been linked to waterlogging responses in barley in other transcriptomic analyses (Borrego-Benjumea *et al*., 2020; Luan *et al*., 2023; Miricescu *et al*., 2023). In the first global transcriptomic study of barley root tissue under waterlogging stress, Borrego-Benjumea *et al*. (2020) also identified enriched GO terms in the upregulated gene set for metabolism (carbohydrate, glycolytic and nucleoside), general enrichment for ‘oxidoreductase activity’ and ‘response to chemical’ related GO terms in Yerong root tissue subjected to 72 h of waterlogging stress. Likewise, short-term waterlogging stress in Franklin and TX9425 barley cultivars revealed higher representation of terms in metabolism modulation, catalytic activity and oxidation–reduction processes (Luan *et al*., 2023). This, together with the observed significant overlap with core transcriptional response to waterlogging defined for barley by Miricescu *et al*. (2023), places this research within the established literature on general transcriptomic responses in barley to waterlogging.

Gene enrichment analysis of genes differentially expressed in both cultivars and specific to the 24 h time point further underscored the importance of redox state management, but also highlighted posttranslational modification-related pathways, including phosphorylation state and cell division, as contributors to the early waterlogging stress response. Although their exact role in waterlogging responses is yet unresolved, kinases and modulation of phosphorylation state play a role in many cellular reactions, including regulation of the activity of the hypoxia-responsive protein respiratory burst oxidase H (RBOHH) in rice roots, which was linked to formation of aerenchyma (Yamauchi *et al*., 2017). Kinase activity was also linked to management of glycolytic activity during anoxia via regulation of hexokinases in maize root tips (Bouny and Saglio, 1996). Management of ubiquitination was also identified in enriched pathways for this time point, and waterlogging tolerance in barley has previously been linked to oxygen sensing via *N*-end rule components controlling proteasomal degradation and activation of hypoxia genes (Mendiondo *et al*., 2016). Furthermore, the 24 h unique downregulated genes here were enriched in cell cycle-related GO terms, which might underscore the requirement to manage cell division and cycle processes under stress. Disruption of the cell cycle is associated with stress in *Arabidopsis* (Takahashi *et al*., 2019) and with programmed cell death responses (Zebell and Dong, 2015; Chen *et al*., 2017; Takahashi *et al*., 2019; Burke *et al*., 2023). Given that plants respond actively to stress, cell cycle arrest is plausible, which minimizes use of energy and resources and is potentially related to localized induction of programmed cell death and subsequent aerenchyma formation.

In contrast, gene expression changes unique to the later, 72 h time point, highlighted modifications of cell wall components occurring at this stage (Fig. 7). This is supported by observations from other studies suggesting that morphological changes associated with cell wall structure generally appear after an extended treatment duration (Watanabe *et al*., 2013), including the cell wall modifications after prolonged hypoxia treatment in maize (Gunawardena *et al*., 2001*b*) and suberin deposition observed only after long-term waterlogging stress in rice (Watanabe *et al*., 2013) and under prolonged nutrient deficiency in barley (Chen *et al*., 2019).

### Genetic regulation of aerenchyma formation in barley

Previous studies reported that aerenchyma percentage varies substantially depending on the sampling location within the root (distance from the base or root) (Bouranis *et al*., 2006; Xu *et al*., 2013; Chen *et al*., 2021), but they did not investigate differences in aerenchyma levels observed between roots of the same individual. In the present study, we observed a high intra-individual variation in this trait, demonstrated by large differences in percentage aerenchyma between two adventitious roots sampled per plant at the same distance from the root tip to increase the chances of collecting tissue at a similar developmental stage. Our data underscore that multiple roots should be sampled per plant to achieve an accurate representation of aerenchyma levels observed for a particular genotype, in specific growth and treatment conditions, because often only a single root is sampled per plant in studies on waterlogging responses. We exploited the observed high intra-individual variation in levels of aerenchyma for identification of putative aerenchyma-associated genes through comparisons of transcriptional profiles of roots differing in percentage aerenchyma, sampled from the same individuals within each genotype, time point and treatment.

Using this approach, we isolated 81 candidate aerenchyma-associated genes (based on BaRTv2 transcriptome annotation). This list included genes encoding proteins previously reported in the context of aerenchyma formation in various plant species other than barley, such as three peroxidases, two kinases (calcium-dependent protein kinase 18 and a putative serine/threonine-protein kinase-like protein, CCR3), numerous carbohydrate modification-associated proteins, including a pectinesterase inhibitor 28 protein, xyloglucan endotransglucosylase/hydrolase, expansin, glycosyltransferase family protein 2 and polygalacturonase. This is in line with previous findings of peroxidases being upregulated under hypoxia stress (Mühlenbock *et al*., 2007; Hofmann *et al*., 2020) and during programmed cell death regulation (Graças *et al*., 2020; Zhang *et al*., 2022), cell wall modifications being an important stage of the aerenchyma formation process (Gunawardena *et al*., 2001*b*; Leite *et al*., 2017; Pegg *et al*., 2020) and reported involvement of kinases in aerenchyma regulation (Wany *et al*., 2017; Yamauchi *et al*., 2017). A particularly interesting aerenchyma-associated candidate gene, which was also strongly induced by 24 h waterlogging in both cultivars (log_2_ fold change 20.81 in Franklin and 38.12 in Yerong), encodes an S-type anion channel, SLAH3 (SLOW ANION CHANEL1 HOMOLOG3). In *Arabidopsis*, SLAH3 was linked to immune responses (Liu *et al*., 2019), control of rapid stomatal closure (A. Zhang *et al*., 2016), plasma membrane potential (Liu *et al*., 2023) and regulation of apoplastic reactive oxygen species-induced cell death (Jalakas *et al*., 2021). Furthermore, SLAH3 promotes plant submergence stress responses in *Arabidopsis* by sensing hypoxia-induced cytosolic acidification and mediating root anion efflux (Lehmann *et al*., 2021). The role of SLAH3 in regulation of cell death mediating aerenchyma formation, potentiated by hypoxic conditions, is therefore plausible.

The gene enrichment analysis further validated the list of aerenchyma-associated candidate genes, highlighting terms linked to processes previously reported to be involved in development of this trait. Gene enrichment analyses [performed using: (1) MorexV3 genome IDs and ShinyGO; and (2) best *Arabidopsis* hits of identified aerenchyma-associated genes and PlantRegMap tool] pointed to cell wall modulation, redox state management and regulation of stress and immune responses, all of which were previously studied in the context of aerenchyma formation in a range of plant species (Saab and Sachs, 1996; Gunawardena *et al*., 2001*b*; Mühlenbock *et al*., 2007; Rajhi *et al*., 2011; Steffens *et al*., 2011; Xu *et al*., 2013; Yamauchi *et al*., 2014, 2017; Chen *et al*., 2016; Leite *et al*., 2017; Pucciariello and Perata, 2017; Wany *et al*., 2017; Wany and Gupta, 2018; Armstrong *et al*., 2019; He *et al*., 2019; Ni *et al*., 2019; Basu *et al*., 2020; Pegg *et al*., 2020; Pan *et al*., 2022).

The functional enrichment analysis also highlighted that histone modifications might play a role in aerenchyma formation. This is in line with a study on wheat seminal roots, where blocking histone acetylation was found to impact waterlogging tolerance and aerenchyma formation through changes to cell wall degradation (Li *et al*., 2019).

Finally, the ‘response to karrikins’, small organic compounds that are released during wildfires from burning plant material (Flematti *et al*., 2015), was among terms enriched among isolated aerenchyma-associated genes. A recent study examined the impact of smoke solution on wheat seedlings under waterlogging stress (Komatsu *et al*., 2022). The karrikin-containing smoke solution alleviated the effect of flood stress on leaf growth, altered metabolic signatures associated with amino acids and maintained abundances of photosynthesis-related proteins in wheat plants under flooding stress. The role of the karrikin signalling pathway is also emerging in relationship to plant responses to other abiotic stress conditions, such as heat, cold, salinity and osmotic stress (Wang *et al*., 2018; Abdelrahman *et al*., 2022), and karrikin signalling was found to promote ethylene synthesis to modulate root architecture (Swarbreck, 2021). Considering the known role of ethylene in aerenchyma formation, further studies exploring the role of the karrikin response pathway in this context are warranted.

Additional insights into regulatory networks underlying aerenchyma formation were provided by the constructed clustered GRN based on the best *Arabidopsis* hits of identified aerenchyma-associated genes. This allowed identification of four hub genes that are highly connected and thus likely to have a central regulatory role in the network: DNA damage response gene *DRT100*, cell wall hydrolases *XTH16* and *XTH4*, and a cell wall protein encoding *AT3G08030*. Interestingly, *DRT100* has been implicated previously in the programmed cell death process of senescence (Ghosh *et al*., 2013) and was identified as hypoxia responsive in *Prunus* rootstock microarray analysis (Rubio-Cabetas *et al*., 2018). Likewise, *XTH16* was upregulated by hypoxia in tomato root tissue (Safavi-Rizi *et al*., 2020); while *XTH4* is associated with modulation of cell wall structure in *Arabidopsis* (Kushwah *et al*., 2020) and fluctuates in expression depending on the root location sampled in aerenchyma-forming sugarcane roots, with root segments with largest proportion of aerenchyma tissue having the highest expression levels of XTH4 (Grandis *et al*., 2019). Finally, *AT3G08030* is implicated in remodelling of the cell wall and response to a number of stresses in *Arabidopsis* (Cruz-Valderrama *et al*., 2019) and has been classified as a molecular marker of seed ageing (Garza-Caligaris *et al*., 2012), but has not previously been linked to waterlogging stress or aerenchyma formation.

Subsequently, analysis of upstream transcriptional regulators of aerenchyma formation was carried out using *Arabidopsis* homologues of identified aerenchyma-associated genes. One of the identified transcription factors, ERF73 was shown to be induced by hypoxia in *A. thaliana* roots and to drive ADH expression and metabolic adaptations (Hess *et al*., 2011). Furthermore, the majority of identified transcription factors belonged to the ERF (ethylene response factor) family, a class of transcription factors frequently associated with low-oxygen responses in numerous species (Xu *et al*., 2006; Hattori *et al*., 2009; Licausi *et al*., 2010; Yang, 2014; Phukan *et al*., 2018; Yin *et al*., 2019). Given that blocking of ethylene perception using 1-MCP partly inhibits aerenchyma formation stimulated by ethylene (Ni *et al*., 2019), this highlights the importance of ethylene in modulating aerenchyma formation by this phytohormone signalling. Transcription factors with targets overrepresented amongst the aerenchyma candidate genes were previously associated with drought, such as DREB2B, DREB19 and MYB94 (Nakashima *et al.*, 2000; Jiang *et al.*, 2022), salt (e.g. AT1G75490, DREB19 and DREB26) (Krishnaswamy *et al*., 2011; Akbudak *et al*., 2018; Kim *et al*., 2022) and cold stresses (e.g. CRF4, ERF040, ERF57, ERF104 and GATA16) (Sun *et al*., 2016; Zwack *et al*., 2016; Illgen *et al*., 2020; Zhang *et al*., 2021; Li *et al*., 2023). Although aerenchyma is a key trait for waterlogging tolerance (Mustroph, 2018), it can also be formed in response to other abiotic stresses, such as drought (J. Zhu *et al*., 2010), mechanical impedance (He *et al.*, 1996) and nutrient deficiency (Postma and Lynch, 2011). Likewise, a recent study has determined the significant overlap of transcriptomic signatures between anaerobic and cold stresses in rice (Thapa *et al*., 2023), suggesting that similar regulatory pathways might modulate adaptation to these stresses.

In conclusion, the presented work significantly advances our understanding of waterlogging stress-induced transcriptional signatures in barley root tissue. Furthermore, exploiting the observed intra-individual variation in aerenchyma formation facilitated isolation of candidate genes associated with this trait and characterization of the regulatory pathways underlying aerenchyma formation. We anticipate that the resources generated will support meta-analyses of the response to waterlogging stress across crop species, in addition to future functional studies aimed at development of waterlogging-tolerant barley cultivars.

## Supplementary Material

mcaf104_Supplementary_Data

## Data Availability

RNA sequences and metadata for all root samples used in this publication have been uploaded to the NCBI Sequence Read Archive and are openly available under the Bioproject accession number PRJNA956333 https://dataview.ncbi.nlm.nih.gov/object/PRJNA956333?reviewer=d98qm8ivmv96s40n46i69nu8cj.

## SUPPLEMENTARY DATA

Supplementary data are available at *Annals of Botany* online and consist of the following. Figure S1: hand sectioning experimental set-up. Figure S2: volcano plots of DGE analyses. Figure S3: volcano plots of DGE analyses. Figure S4: volcano plots of DGE analyses. Figure S5: basal gene expression levels in barley cultivars compared with waterlogging-responsive genes. Figure S6: gene ontology (GO) enrichment of Franklin WL response genes. Figure S7: gene ontology (GO) enrichment of Yerong WL response genes. Table S1: RNA sequencing sample information. Table S2: barley gene ID conversion table. Table S3A: transcriptional response of barley roots to 24 and 72 h of waterlogging stress. Table S3B: differentially expressed genes in barley roots with a higher percentage of aerenchyma compared with a lower percentage of aerenchyma. Table S3C: members of aerenchyma-associated genes differentially expressed in waterlogging stress treatments. Table S4A: differentially expressed genes in barley roots to 24 and 72 h of waterlogging stress compared with hypoxia-responsive genes from Miricescu *et al*. (2023). Table S4B: comparison of waterlogging-responsive genes with hypoxia-responsive genes from Miricescu *et al*. (2023). Table S5: aerenchyma-associated gene list transcription factor enrichment.
