# Supplementary material for: Transcriptional signatures associated with waterlogging stress responses and aerenchyma formation in barley root tissue
Source: Ann Bot. 2025 Jun 18;136(5-6):1175–91. doi: 10.1093/aob/mcaf104 (PMC12682839; doi:10.1093/aob/mcaf104)
Supplement: mcaf104_Supplementary_Data [file mcaf104_supplementary_data.zip › aob-24822-s01.docx]

25^th^ March 2025

Dear Editor and Panel of Reviewers,

we are pleased that the previously submitted version of the manuscript has alleviated the key reviewers’ concerns. We would like to kindly thank the editor for their additional suggestions, that as outlined below, are addressed in the current version of the manuscript. Collectively, we are thankful for the constructive review process that made the our manuscript clearer, easier to follow, and ensured that it will be of interest to the broad plant science community.

Reviewers’ comments:

Referee #1

Authors are advised to go through the manuscript carefully and italic all scientific names and genes.
Two questions still remain unanswered-
1. Have you validated those genes identified from previous studies in your material in response to waterlogging? If not, why?
2.Have you validated your RNAseq data of selected DEGs through qRT-PCR? if not, why?

Response: We would like to take this opportunity to thank you for time spent on our work and your suggestions that helped us to improve it. We confirm that we have went through the current version carefully to ensure that scientific. Regarding the two questions outstanding:

Q1.	To validate our results against other studies on waterlogging, we compared the transcriptional response induced by waterlogging stress in our study with the list of core waterlogging-response genes recently identified through analysis of 21 cultivars by Miricescu et al (2023). Reassuringly, this comparison revealed a significant overlap (Table S4A), with the same direction of gene regulation.

Q2.	We did not validate the RNAseq data through quantitative PCR. We decided against this based on: (1) the cost of additional qPCR analysis and (2) the strong literature evidence (see references listed below for examples) for extremely close correlation between qPCR and RNAseq data, and for robustness of RNA sequencing. As argued by Coenye (2021), for RNA-seq studies based on a sufficient number of biological replicates, the added value of validating them with qPCR is likely to be low. Having said that, in the future, it would be great to re-validate the findings using separate cohort of samples (e.g. roots differing in aerenchyma levels from range of different barley cultivars) but at the moment carrying out extra experimental work is beyond the scope of current project due to personnel and funding constraints.

Coenye T. 2021 Do results obtained with RNA-sequencing require independent verification?. *Biofilm* 13(3): 100043. doi: 10.1016/j.bioflm.2021.100043.

Everaert, C., Luypaert, M., Maag, J.L.V. et al. 2017. Benchmarking of RNA-sequencing analysis workflows using whole-transcriptome RT-qPCR expression data. *Sci Rep* 7: 1559. https://doi.org/10.1038/s41598-017-01617-3

Griffith M, Griffith OL, Mwenifumbo J, Goya R, Morrissy a S, et al. (2010) Alternative expression analysis by RNA sequencing. Nat Methods 7: 843–847. doi:10.1038/nmeth.1503.

Asmann YW, Klee EW, Thompson EA, Perez E a, Middha S, et al. (2009) 3’ tag digital gene expression profiling of human brain and universal reference RNA using Illumina Genome

Analyzer. BMC Genomics 10: 531. doi:10.1186/1471-2164-10-531.

Wu AR, Neff NF, Kalisky T, Dalerba P, Treutlein B, et al. (2014) Quantitative assessment of single-cell RNA-sequencing methods. Nat Methods 11: 41–46. doi:10.1038/nmeth.2694.

Shi Y, He M (2014) Differential gene expression identified by RNA-Seq and qPCR in two sizes of pearl oyster (Pinctada fucata). Gene 538: 313–322. doi:10.1016/j.gene.2014.01.031.

Wang Z, Gerstein M, Snyder M (2009) RNA-Seq: a revolutionary tool for transcriptomics. Nat Rev Genet 10: 57–63. doi: 10.1038/nrg2484.

Hughes TR (2009) “Validation” in genome-scale research. J Biol 8: 3. doi:10.1186/jbiol104.

Referee #2's Review for 24822R:

Comments for the Author (Required):
No additional comments or suggestions at this time.

Response: We would like to take this opportunity to thank the reviewer 2 for their time and support.

Further comments from the editor:

General comments:

-Somewhere in the manuscript, you should mention you are studying lysigenous aerenchyma (rather than schizogenesis aerenchyma).

Response: Thanks, we have included this in the revised ms.

-Can we learn anything about what we know about the genetic control of aerenchyma in other crops and apply it to barley? We know some of the important genes in maize and rice and I would guess some of these are conserved in barley.

Response: This is of course a really good point, we agree that a comparative study focused on genes linked to aerenchyma formation across key crops (including both constitutive and inducible forming species) would be timely in light of the recent progress in this area. This considered, we think that this may be beyond the scope of this manuscript which focuses on transcriptional regulation of aerenchyma formation in barley. Please note, however, that in our Discussion section we do extensively comment on the aerenchyma associated-genes and pathways from the current manuscript in context of their known involvement in aerenchyma formation and programmed cell death based on prior studies.

-In the methods (and in figure 1), please describe the control treatment better. Also, due to the waterlogging treatment, did the waterlogged plants receive more nutrients? Without information on the control treatment it is difficult to tell, as in the waterlogging treatment, you filled the bags with Hoagland solution.

We clarified the watering schedule of control plants in the methods section and figure legend of figure 1. Use of Hoagland solution was essential as plants were grown in vermiculate as growth substrate. The waterlogging schedule of control plants (quarter strength Hoagland solution three times a week) prevented drying out of the vermiculite at any stage in the control treatments. It is possible that there were some differences in nutrient available to control and waterlogged plants the waterlogged plants, we did not measure the nutrient uptake/availability.

-How did you deal with tillers in your study? Did you treat adventitious roots from tillers the same as from the main stem?

We treated adventitious roots from tillers the same as the main stem.

-In the methods section, please outline how you calculated aerenchyma %. Some literature uses the area of aerenchyma relative to the root-cross-sectional area, others relative to the cortical area.

Thanks for pointing this out, we have clarified this in the revised ms.

-In all of the figures that have the y axis 'duration (days)' please indicate if this is the duration of growth or duration of the waterlogging treatment.

We have amended the revised ms to include the statement “Duration corresponds to duration of treatment.” in the legends for Figures 2 and 3.

-The title and entire manuscript suggest you are focusing on the genetic control of aerenchyma, but how can you be sure you are not tracking down genes for other waterlogging responses?

The think we acknowledge this in title of manuscript – as it indicates that transcriptional responses associated with both waterlogging, and aerenchyma formation, were analysed (“**Transcriptional signatures associated with waterlogging stress responses and aerenchyma formation in barley root tissue”)**. Likewise, in the main body we specify which figures and analyses characterize waterlogging response (Fig 4, 5 – comparisons between waterlogged and controlled samples) and which ones are focused on isolating genes putatively driving differences in aerenchyma formation (Fig 6) through harnessing intraindividual variability in aerenchyma levels to identify genes differentially expressed between roots from the same individuals differing in aerenchyma level. Reflecting on your comment we have decided to amend the title of Figure 6 to make it clearer.

-I think the difference between L and H aerenchyma in the gene expression results needs to be highlighted and explored more, or tone down the emphasis on aerenchyma in the rest of the manuscript. Or maybe I just do not understand Figure 6 panel A properly. In the red box, it appears that it indicates overlapping DEGs between L and H aerenchyma, not unique genes between them? Don't you want to explore the unique genes between high and low aerenchyma samples? I see that there are 81 DEGs unique to C_H_AE vs L AE in Yerong, but this is not a waterlogging treatment? The legend or figure needs some clarification.

Thanks for these comments. We have amended the title of figure 6 to make it clear that the aerenchyma associated genes were isolated exploiting **intra-**individual variability in aerenchyma formation. We also clarify now in the figure legend that “Red box indicates aerenchyma-associated DEGs present in common to at least two out of eight of the high vs low percentage aerenchyma comparison groups (81 DEGs total).” The fact that there was 81 aerenchyma associated genes (inside the red box) and 81 DEGs unique to C_H_AE vs L AE in Yerong is just a coincidence (these two are not related).

Specific comments:

Line 93: Radial oxygen loss barriers are also a very important anatomical adaptation to waterlogging stress.

Thanks, we have included reference to ROL in the revised ms.

Line 213: Aerenchyma 'pockets' should be changed to 'lacunae'

Done, amended.

Line 523: double period punctuation '. .'

This is amended.

Figure 3: Change 'shoot growth' in panel A to shoot height to match description and terminology in the methods and to avoid confusion with biomass.

We have not changed this in Figure 2 as the measurement reported describes the difference in shoot height from initiation of the waterlogging treatment and at each day following waterlogging. In this way, changing to shoot height would be misreporting as the shoot height is greater overall than the difference in growth.
